# Methods for calculating confidence and credible intervals for the residual between-study variance in random effects meta-regression models

**DOI:** 10.1186/1471-2288-14-103

**Published:** 2014-09-06

**Authors:** Dan Jackson, Rebecca Turner, Kirsty Rhodes, Wolfgang Viechtbauer

**Affiliations:** 1 MRC Biostatistics Unit, Cambridge, UK; 2 Department of Psychiatry and Psychology School for Mental Health and Neuroscience Faculty of Health, Medicine, and Life Sciences Maastricht University, P.O. Box 616 (VIJV1), 6200 MD, Maastricht, The Netherlands

**Keywords:** Heterogeneity, Informative priors, Meta-regression, Quadratic forms

## Abstract

**Background:**

Meta-regression is becoming increasingly used to model study level covariate effects. However this type of statistical analysis presents many difficulties and challenges. Here two methods for calculating confidence intervals for the magnitude of the residual between-study variance in random effects meta-regression models are developed. A further suggestion for calculating credible intervals using informative prior distributions for the residual between-study variance is presented.

**Methods:**

Two recently proposed and, under the assumptions of the random effects model, exact methods for constructing confidence intervals for the between-study variance in random effects meta-analyses are extended to the meta-regression setting. The use of Generalised Cochran heterogeneity statistics is extended to the meta-regression setting and a Newton-Raphson procedure is developed to implement the Q profile method for meta-analysis and meta-regression. WinBUGS is used to implement informative priors for the residual between-study variance in the context of Bayesian meta-regressions.

**Results:**

Results are obtained for two contrasting examples, where the first example involves a binary covariate and the second involves a continuous covariate. Intervals for the residual between-study variance are wide for both examples.

**Conclusions:**

Statistical methods, and R computer software, are available to compute exact confidence intervals for the residual between-study variance under the random effects model for meta-regression. These frequentist methods are almost as easily implemented as their established counterparts for meta-analysis. Bayesian meta-regressions are also easily performed by analysts who are comfortable using WinBUGS. Estimates of the residual between-study variance in random effects meta-regressions should be routinely reported and accompanied by some measure of their uncertainty. Confidence and/or credible intervals are well-suited to this purpose.

## Background

Meta-analysis is well established in medical statistics and meta-regression models [1-3] are becoming increasingly popular [4]. Here, study level covariates, sometimes referred to as moderator variables, are included in the model. A fixed effect meta-regression model, where the residual between-study variance is taken to be zero, results from the assumption that these covariates explain all between-study heterogeneity. Under this strong assumption, exact inference for the covariate effects is straightforward. This analysis can be performed using output from fitting standard weighted linear regression models, whilst ensuring that the reported standard errors are adjusted, as explained by Sharp and Thompson [2].

This fixed effect assumption is however generally implausible, because in most applications we anticipate the existence of residual between-study heterogeneity (i.e. between-study heterogeneity that is not explained by the covariates). Hence, the use of random effects meta-regression models (also called mixed-effects meta-regression models) is typically recommended [2]. This model collapses to a fixed effect meta-regression only if the estimated residual between-study variance is zero. The standard approach for making inferences about the covariate effects using the random effects meta-regression model involves initially estimating the residual between-study variance and then treating this estimate as the true value, but a refined method has also been suggested [1]. This standard approach results in straightforward inference for the covariate effects [2], but the resulting inferences are approximate and reasonably large numbers of studies are needed for this approach to provide accurate inference [5]. Furthermore, meta-regression models are subject to further statistical issues and limitations [6].

The covariate effect parameters from a random effects meta-regression are usually of primary interest but the residual between-study variance is also of interest. This is because its magnitude describes the extent of the unexplained variation in the studies’ results. However, the uncertainty in the estimated between-study variance in random effects meta-analyses is usually considerable [7,8] and this can also be expected to be the case in meta-regression models unless very many studies contribute to the analysis. Hence interpreting point estimates of the residual between-study variance without any regard to their uncertainty, which is quite common practice, could be misleading.

The main contributions of this paper are to show that two recently proposed exact frequentist methods for random effects meta-analysis (generalised *Q* statistics and the *Q* profile method [8,9]) may be extended to meta-regression models, and to suggest how informative distributions for the between-study variance in Bayesian random effects meta-analysis [10] can also be used in the meta-regression setting. Our hope is that meta-analysts will consider using these methods to perform interval estimation for the residual between-study variance in their random effects meta-regressions and that they will find these intervals to be a useful aid to inference. However it is important to recognise that the two frequentist methods are only exact under the random effects meta-regression model. Hence the exactness of the confidence intervals for both examples below is brought into question, because the random effects model only provides an approximation for real data such as these. Some may therefore describe our confidence intervals as ‘non-approximate’ or ‘small sample’ confidence intervals, in order to avoid any connotations of the word ‘exact’.

Although the proposed extension of the *Q* profile method for meta-regression has previously been described, Viechtbauer’s account [11] does not provide proof that this procedure is guaranteed to produce a confidence interval (instead of a confidence set that need not be an interval). Furthermore, the proof of the result that ensures this by Panityakul *et al*. [12] involves quite sophisticated matrix theorems and calculations. In this paper we explain why both the proposed frequentist methods provide confidence intervals for the residual between-study variance. We also provide an alternative and, in our opinion, more elementary proof of the result given by Panityakul *et al*. As we explain below, our proof provides an easily computed derivative that can be used in a Newton-Raphson procedure for implementing the *Q* profile method in practice. To our knowledge, the two frequentist methods proposed in this paper are the only methods currently available that provide confidence intervals for the residual variance with exactly the nominal coverage probability under the random effects model for meta-regression. However, alternative, but approximate, methods for obtaining confidence intervals are also available in the meta-analysis setting [9] that could also be extended to meta-regression.

### The random effects model for meta-regression

The random effects meta-regression model assumes that $Y_{i}|x_{i}∼N(x_{i}\beta ,\sigma_{i}^{2}+\tau^{2})$, where *Y*_*i*_ is the estimated effect from the *i*th study, *i*=1,2,⋯*n*, *x*_*i*_ is the 1×*p* row vector of covariates associated with this study and *β* is the vector of regression parameters of interest. Unless an intercept free regression is required, the first ‘covariate’ in each study is taken to be one to include the intercept. The parameter *τ*^2^ is the residual between-study variance that describes the variation in the results that is not explained by the covariates. The within-study variances $\sigma_{i}^{2}$ are estimated in practice but are treated as fixed and known in the analysis. The matrix formulation of this standard model is

(1) $$Y|X∼N\left(X\beta ,\Delta +\tau^{2}I\right)$$

where ***Y*** is a column vector containing the *Y*_*i*_, ***X*** is the *n*×*p* design matrix (sometimes referred to as the model matrix) whose *i*th row is ***x***_*i*_, $\Delta =\text{diag}(\sigma_{i}^{2})$ (i.e. ***Δ*** is the diagonal matrix containing the $\sigma_{i}^{2}$) and ***I*** is the *n*×*n* identity matrix. We will also define and make frequent use of ***Σ*** = ***Δ*** +*τ*^2^ ***I***. Although we use the conventional regression terminology of a ‘design matrix’ when referring to ***X***, we agree with Thompson and Higgins [6] that meta-regressions have the same disadvantages as observational epidemiological investigations and that the covariates used in meta-regressions are rarely, if ever, chosen by design.

All the methods that follow are for performing interval estimation of *τ*^2^ under the assumptions of the random effects model for meta-regression. This model makes a number of important assumptions, such as normal distributions, independent outcomes, fixed and known within study distributions and linear covariate effects. Assumptions such as these are usually approximations in practice. Hence the extent to which model (1) is an approximation should be taken into account when interpreting the intervals for *τ*^2^ below. Alternative types of inference for the residual between-study variance parameter, such as testing for the presence of unexplained between-study heterogeneity and measuring its impact, can be obtained from the intervals presented in this paper. We return to these possibilities in the discussion.

## Methods

In this section we develop two exact frequentist methods for computing confidence intervals for *τ*^2^ and we also present our proposal for Bayesian meta-regressions using informative prior distributions for this parameter. By ‘exact’ we mean, under the random effects model for meta-regression (1), that these two frequentist methods provide confidence intervals with exactly the nominal coverage probability (subject to the way we interpret empty confidence intervals, see below). A disadvantage of these methods is that they are not based on sufficient statistics and so have not been shown to possess any optimality properties. We assume throughout that the design matrix is of full rank, so that all parameters are identifiable. In practice we suggest that, as a minimum, *n*=10 is needed to fit a meta-regression with a single covariate effect. Even larger *n* will be required in order to estimate the effect of multiple covariates in the same meta-regression model.

### Generalised Cochran heterogeneity statistics for meta-regression

The conventional *Q* statistic for meta-regression is

(2) $$Q=\overset{n}{\underset{i=1}{\sum }}w_{i}{\left(y_{i}-{ŷ}_{i}\right)}^{2}$$

where $w_{i}=\sigma_{i}^{-2}$ and ${ŷ}_{i}$ is the fitted value for the *i*th study from the fixed effects model, where *τ*^2^=0. That is ${ŷ}_{i}=x_{i}{\hat{\beta }}_{F}$ where ${\hat{\beta }}_{F}={\left(X^{t}WX\right)}^{-1}X^{t}WY$ and ***W*** =diag(*w*_*i*_)= ***Δ***^-1^. Under the null hypothesis *H*_0_:*τ*^2^=0, *Q* follows a $\chi_{n-p}^{2}$ distribution and so may be used as a test statistic.

DerSimonian and Kacker [13] proposed a generalised version of *Q* in the special case of meta-analysis and where the only ‘covariate’ is the intercept. They proposed using an arbitrary set of fixed positive constants *a*_*i*_ instead of *w*_*i*_ when computing (2). Jackson [8] showed that this can be used to compute confidence intervals for the between-study variance in random effects meta-analyses. An obvious and more general version of DerSimonian and Kacker’s heterogeneity statistic for meta-regression is

(3) $$Q_{a}=\overset{n}{\underset{i=1}{\sum }}a_{i}{\left(y_{i}-{ŷ}_{i}\right)}^{2}$$

where the *a*_*i*_ are arbitrary positive constants and ${ŷ}_{i}$ is now ${ŷ}_{i}=x_{i}{\hat{\beta }}_{a}$, where ${\hat{\beta }}_{a}={\left(X^{t}AX\right)}^{-1}X^{t}AY$ and ***A*** =diag(*a*_*i*_). If *a*_*i*_=*w*_*i*_ for all *i*, or equivalently ***A*** = ***W***, then *Q*_*a*_ in equation (3) reduces to the usual heterogeneity statistic (2). We will develop the theory in terms of *Q*_*a*_ and so include the conventional *Q* as a special case.

In order to derive the properties of (3) we write this in matrix form. We have that

$$Y-\hat{Y}=\left(I-X{\left(X^{t}AX\right)}^{-1}X^{t}A\right)Y$$

and (3) can be written as

$$Q_{a}={\left(Y-\hat{Y}\right)}^{t}A\left(Y-\hat{Y}\right).$$

After a little manipulation we can write

(4) $$Q_{a}=Y^{t}BY$$

where ***B*** = ***A***-***A*** ***X*** (***X***^*t*^ ***A*** ***X***)^-1^ ***X***^*t*^ ***A***.

Next, essentially following Biggerstaff and Jackson [7] and Jackson [8], let **Z** denote a standard *n* dimensional multivariate normal vector. Noting that *Q*_*a*_ is ‘location invariant’ (by this we mean that we can write **Y**= ***X*** ***β*** +**Σ**^1/2^**Z** in equation (4), where **Z** is standard multivariate normal, and the location ***X*** ***β*** cancels in the computation of *Q*_*a*_), we can obtain

$$Q_{a}=Y^{t}BY\overset{d}{=}Z^{t}\Sigma^{1/2}B\Sigma^{1/2}Z=Z^{t}SZ,$$

defining **S**=**Σ**^1/2^**BΣ**^1/2^. **B** is symmetric and hence so is **S**. Following the same procedure described by Biggerstaff and Jackson [7] and Jackson [8], writing **S** in terms of its spectral decomposition, we obtain

(5) $$Q_{a}\overset{d}{=}\overset{n-p}{\underset{i=1}{\sum }}\lambda_{i}(S)\chi_{1,i}^{2}$$

where $\chi_{1,i}^{2}$ are mutually independent chi-squared random variables with 1 degree of freedom and *λ*_1_(**S**)≥*λ*_2_(**S**)≥⋯≥*λ*_*n*_(**S**) are the ordered eigenvalues of **S**. Because we take the covariates, the within-study variances $\sigma_{i}^{2}$ and the *a*_*i*_ as fixed constants, from (5) we have that the only unknown parameter that the distribution of *Q*_*a*_ depends on is *τ*^2^. The summation in (5) extends to *n*-*p*, rather than *n*, because *p* of the eigenvalues of **S** are zero. This is because ***S*** = ***Σ***^1/2^ ***A***^1/2^(***I***-***H***) ***A***^1/2^ ***Σ***^1/2^, where ***H*** is the hat matrix for the regression where the design matrix is ***A***^1/2^ ***X***. Because ***A*** and ***Σ*** are diagonal, and because the eigenvalues of ***C*** ***D*** are those of ***D*** ***C*** when ***C*** and ***D*** are both square matrices [14], p 57, the eigenvalues of ***S*** are those of ***Σ***^1/2^ ***A***^1/2^(***I***-***H***) ***A***^1/2^ ***Σ***^1/2^ or equivalently those of (***I***-***H***) ***A*** ***Σ***. Then, because (***I***-***H***) and ***A*** ***Σ*** are positive semi-definite symmetrical matrices, Theorem 8.12 from Zhang [14], p 274, implies that *λ*_*i*_(**S**)=*λ*_*i*_((***I***-***H***) ***A*** ***Σ***)≤*λ*_*i*_(***I***-***H***)*λ*_1_(***A*** ***Σ***). *λ*_1_(***A*** ***Σ***)>0 and, because *Q*_*a*_ is non-negative, the eigenvalues of **S** are also non-negative; *p* eigenvalues of (***I***-***H***) are zero and hence so are *p* eigenvalues of **S**, as stated.

#### Obtaining confidence intervals numerically

The cumulative distribution function of *Q*_*a*_ is decreasing in *τ*^2^. This is because the eigenvalues of ***S*** are increasing in *τ*^2^. This can be shown in a similar way to the meta-analysis setting as explained by Jackson [8]. The eigenvalues of **S** are those of ***Σ***^1/2^ ***A***^1/2^(***I***-***H***) ***A***^1/2^ ***Σ***^1/2^ as noted above. Consider a larger between-study variance, so that ***Σ*** becomes ***Σ*** ***M*** and ***Σ***^1/2^ becomes ***Σ***^***1/2***^ ***M***^1/2^, where **M** is a diagonal matrix in which all diagonal entries and hence all eigenvalues are also greater than one. Then Theorem 8.12 from Zhang [14], p 274, implies that *λ*_*i*_ (***A***^1/2^ ***Σ***^***1/2***^ ***M***^***1/2***^(***I***-***H***) ***A***^1/2^ ***Σ***^***1/2***^ ***M***^***1/2***^)=*λ*_*i*_(***A***^1/2^ ***Σ***^***1/2***^(***I***-***H***) ***A***^1/2^ ***Σ***^***1/2***^ ***M***)≥*λ*_*i*_((***A***^1/2^ ***Σ***^***1/2***^(***I***-***H***) ***A***^1/2^ ***Σ***^***1/2***^))*λ*_*n*_(**M**), which means that the larger between-study variance has resulted in larger eigenvalues of **S**. Hence these eigenvalues are increasing in *τ*^2^ as stated.

This ensures that confidence intervals (rather than confidence sets) for *τ*^2^ can be obtained in the way described by Jackson [8] for meta-analysis, using the distributional result in (5) and test inversion (eg Casella and Berger [15], section 9.2.1). This means that $\tau_{J}^{2}$ is accepted by the two-tailed hypothesis test, and so lies in the corresponding confidence set, if and only if

(6) $$P\;\left(Q_{a}\geq q_{a};\tau^{2}=\tau_{J}^{2}\right)\geq \alpha /2$$

and

(7) $$P\;\left(Q_{a}\leq q_{a};\tau^{2}=\tau_{J}^{2}\right)\geq \alpha /2$$

where *q*_*a*_ is the observed value of *Q*_*a*_ and the coverage probability is (1-*α*). As noted by Jackson [8], alternative values to *α*/2 are possible in (6) and (7), but we will use ‘equal tailed’ two-sided confidence intervals. If no $\tau_{J}^{2}$ satisfies (7) then we have two main choices. The first option is to follow Jackson [8] in providing an interval of [0,0] that increases the coverage probability by *α*/2 when *τ*^2^=0 which is therefore conservative. The second option is to provide an empty set as suggested by Viechtbauer [9] in relation to the *Q* profile method below. Providing the empty set retains the nominal coverage probability for all *τ*^2^ but means that the analyst is faced with the task of explaining why the empty set is appropriate to practitioners. We leave it to the reader to decide which convention they prefer. Since the empty set is itself an interval, either convention can be described as providing confidence intervals. A possible third convention, as discussed by Jackson [8], would be to refrain from giving a confidence interval in such instances and instead conclude that the interval is undefined. This statement would be accompanied with a conclusion like ‘the data appear to be highly homogenous’ or that ‘the interval estimation fails’.

Because the cumulative distribution function of *Q*_*a*_ is decreasing in *τ*^2^, we can easily perform numerical searches to obtain the confidence interval limits using Farebrother’s algorithm [16] to evaluate the distribution function of *Q*_*a*_ implied by (5). Since, as in the *Q* profile method below, the confidence intervals are based upon an exact (under the assumptions of the model) distributional result, exact confidence intervals are obtained, subject to the [0,0] or empty set caveat discussed above. An *R* function is provided in the Additional files 1, 2 and 3 that produces confidence intervals using this method, where empty confidence sets are given as [0,0]. This function has now been implemented in the *metafor* package and code for use with this package is also provided in the Additional files 1, 2 and 3.

#### Choosing the ‘weights’

One issue when using this procedure is that it involves choosing a set of ‘weights’ *a*_*i*_. Jackson [8] discusses possible approaches for this. In the absence of information about the likely magnitude of the between-study variance, Jackson [8] suggested using *a*_*i*_=1/*σ*_*i*_ but practitioners may prefer to use the conventional fixed effects weights $a_{i}=1/\sigma_{i}^{2}$ because these may be more familiar. An investigation into the optimal choice of weights, as undertaken in a simulation study by Jackson [8], could provide suitable further work but similar results to those in the meta-analysis (no covariates) scenario may reasonably be anticipated: that is, weights that reflect the magnitude of the true residual heterogeneity (equal to the reciprocal of the true total variances) can be expected to perform well.

#### Moments of generalised Cochran heterogeneity statistics

The above method of computing confidence intervals using generalised Cochran heterogeneity statistics requires the use of Farebrother’s algorithm and iterative methods to find the bounds of the confidence interval. In this section we show how to use the moments of generalised Cochran heterogeneity statistics to produce a point estimate of the residual between-study variance and a much simpler method for quantifying the uncertainty in this point estimate. The point estimate of *τ*^2^ proposed in this section is a natural estimate to accompany the confidence interval obtained using a generalised Cochran heterogeneity statistic.

In Appendix 1, we show that the expectation of the generalised Cochrane heterogeneity statistic is

(8) $$E(Q_{a})=\text{tr}\left(B\Delta \right)+\text{tr}(B)\tau^{2}$$

where tr (·) denotes the trace of a matrix. Alternatively, E(*Q*_*a*_) can be evaluated numerically as $\overset{n-p}{\underset{i=1}{\sum }}\lambda_{i}(S)$.

Hence (8) provides a moments based estimator of *τ*^2^ upon replacing E(*Q*_*a*_) with *Q*_*a*_, solving the resulting equation and truncating to zero if the estimate is negative. Equation (8) reduces to the usual formula [1] for the expectation of *Q* when *a*_*i*_=*w*_*i*_ for all *i*, or equivalently that **A**= ***W***, so that the conventional moments based estimator is obtained as this special case. Using (8) to produce a point estimate of *τ*^2^ using the method of moments in this way, we obtain an ‘untruncated’ (not set to zero if negative) estimate

(9) $${\hat{\tau }}_{u}^{2}=\frac{Q_{a}-\text{tr}\;(B\Delta )}{\text{tr}\;(B)}.$$

The estimator ${\hat{\tau }}_{u}^{2}$ is unbiased. Upon truncating ${\hat{\tau }}_{u}^{2}$ to zero if it is negative, which introduces positive bias, we obtain a generalisation of the estimator using DerSimonian and Kacker’s [13] general method of moments for meta-analysis. This estimator is itself a generalisation of the method proposed by DerSimonian and Laird [17]. Since the entries of **B** are fixed constants, we have

(10) $$\text{Var}\left({\hat{\tau }}_{u}^{2}\right)=\frac{\text{Var}\;(Q_{a})}{{\left(\text{tr}\;(B)\right)}^{2}}.$$

Using standard properties of quadratic forms involving normal distributions, (e.g., Searle [18], page 57, Corollary 1.2), we have that

(11) $$\begin{array}{c} \text{Var}\;(Q_{a})=\;2\text{tr}\;(B\Sigma B\Sigma )=2\text{tr}(\;B\Delta B\Delta )+4\tau^{2}\text{tr}\;(B\Delta B)\\ \;+2\tau^{4}\text{tr}\;\left(B^{2}\right) \end{array}$$

This formula reduces to equation (7) from Biggerstaff and Tweedie [19] for meta-analyses and the standard weights if *a*_*i*_=*w*_*i*_. Alternatively, Var(*Q*_*a*_) can be obtained as $2\overset{n-p}{\underset{i=1}{\sum }}\lambda_{i}^{2}(S)$. Together (10) and (11), after taking the square root, provide the standard error and hence a measure of the uncertainty in the ‘untruncated’ estimate ${\hat{\tau }}_{u}^{2}$. However, the distribution of *Q*_*a*_, and hence the distribution of ${\hat{\tau }}_{u}^{2}$, is usually very skewed in typical meta-analytic datasets with few studies. Hence using a normal approximation for these statistics is generally inadequate. For this reason we do not propose that confidence intervals are obtained using ${\hat{\tau }}_{u}^{2}$, its standard error and a normal approximation, but the standard error of ${\hat{\tau }}_{u}^{2}$ could still be used to give an indication of the uncertainty in this point estimate by those who do not have access to Farebrothers’ algorithm and/or iterative methods. When ${\hat{\tau }}_{u}^{2}>0$, a normal approximation on the log scale might be expected to perform better because a log transformation would counteract the skewness of *Q*_*a*_ but the resulting confidence interval could then not include zero.

#### Using weights equal to the reciprocal of the estimated total variances

Methods for estimating *τ*^2^ have been suggested above and it is tempting to consider using weights $a_{i}=1/(\sigma_{i}^{2}+{\hat{\tau }}^{2})$, where ${\hat{\tau }}^{2}$ is some estimate of *τ*^2^. Jackson [8] found that this idea performed well in random effects meta-analyses using the DerSimonian and Laird [17] estimator. Weights of this form reflect the variation in the sample of study estimates, and can be expected to provide precise estimation (and so shorter confidence intervals). These weights mean that the conventional weights for making inferences about the overall effect are also used for making inferences about the residual between-study variance. However this choice of weights invalidates the theory because the weights are now random variables and are no longer fixed constants. Jackson [8] found that this problem was of little practical consequence in the context of meta-analysis and this idea warrants further investigation in both the meta-analysis and meta-regression scenarios.

### The Q profile method for meta-regression

The *Q* profile method for meta-analysis has recently been proposed [9,20,21]. Viechtbauer [11] and Panityakul *et al*. [12] subsequently explained how to extend this method to meta-regression models in the way we also describe below. This method is based on the *Q* profile statistic given in equation (12) below and is not based on the profile likelihood. The *Q* profile method has been implemented in the *R* package *metafor* for both meta-analyses and meta-regressions. In this paper we show that the method proposed by Viechtbauer [11] and Panityakul *et al*. [12] for meta-regression is guaranteed to provide confidence intervals, that is, confidence sets that are not intervals are never produced. Viechtbauer [11] implicitly assumes that this is the case. The previous proof [12] that the function *Q*(*τ*^2^) below is decreasing in *τ*^2^ is sophisticated, so the main contribution of this paper concerning this particular method is to prove this result in a simpler and more direct way. We do this in a similar way to Knapp *et al.*[21], who prove this result in the simpler setting of the random effects model for meta-analysis (no covariates).

In order to describe the *Q* profile method for meta-regression, we define ${ŷ}_{i}(\hat{\beta }(\tau^{2}))$ as the fitted value of *Y*_*i*_ from the true meta-regression model. This means that ${ŷ}_{i}\left(\hat{\beta }\left(\tau^{2}\right)\right)=x_{i}\hat{\beta }\left(\tau^{2}\right)$, where $\hat{\beta }\left(\tau^{2}\right)={\left(X^{t}\Sigma^{-1}X\right)}^{-1}X^{t}\Sigma^{-1}Y$.

The *Q* profile method is then based on

(12) $$Q\;\left(\tau^{2}\right)=\overset{n}{\underset{i=1}{\sum }}\frac{{\left\{Y_{i}-{ŷ}_{i}\left(\hat{\beta }(\tau^{2})\right)\right\}}^{2}}{\sigma_{i}^{2}+\tau^{2}}∼\chi_{n-p}^{2}$$

where we use (12) as a pivot to test the null hypothesis that the true value of the residual between-study variance is equal to *τ*^2^. If $\chi_{\left(n-p,\alpha /2\right)}^{2}\leq Q(\tau^{2})\leq \chi_{\left(n-p,1-\alpha /2\right)}^{2}$, where $\chi_{\left(n-p,\alpha /2\right)}^{2}$ and $\chi_{\left(n-p,1-\alpha /2\right)}^{2}$ denote the *α*/2 and 1-*α*/2 quantiles of the $\chi_{n-p}^{2}$ distribution respectively, then we accept (or fail to reject) the null hypothesis that the true residual between-study variance is equal to *τ*^2^ using a significance level of *α*. Otherwise, we reject this hypothesis.

The confidence set for the residual between-study variance, with (1-*α*) coverage probability, is again obtained by test inversion. The confidence set for *τ*^2^ contains all the values of *τ*^2^ that are accepted by the hypothesis test resulting from (12).

#### Ensuring that confidence intervals are obtained

As previously noted [12], the condition that the derivative *dQ*(*τ*^2^)/*dτ*^2^ is negative is sufficient to show that the *Q* profile method for meta-regression is guaranteed to provide confidence intervals. In Appendix 2, we show that

(13) $$\frac{\text{dQ}\left(\tau^{2}\right)}{d\tau^{2}}=-\overset{n}{\underset{i=1}{\sum }}\frac{{\left\{Y_{i}-{ŷ}_{i}\left(\hat{\beta }(\tau^{2})\right)\right\}}^{2}}{{\left(\sigma_{i}^{2}+\tau^{2}\right)}^{2}}<0$$

which means that *Q*(*τ*^2^) is decreasing in *τ*^2^. This is a generalisation of the result provided in Appendix 2 of Hartung and Knapp [20], that Knapp *et al.*[21] refer to when establishing that the *Q* profile method for meta-analysis is guaranteed to provide confidence intervals. The form of the derivative in (13) is considerably more straightforward than the expression previously given [12].

#### Obtaining confidence intervals numerically

From (13) we have that *Q*(*τ*^2^) is decreasing in *τ*^2^. If $Q(0)<\chi_{\left(n-p,\alpha /2\right)}^{2}$ then no *τ*^2^ satisfies $\chi_{\left(n-p,\alpha /2\right)}^{2}\leq Q(\tau^{2})\leq \chi_{\left(n-p,1-\alpha /2\right)}^{2}$, and an empty confidence set is obtained. This occurs when the data appear to be highly homogenous, and we can either follow Knapp *et al.*[21] and Jackson [8] by interpreting these empty sets as providing confidence intervals of [0,0], or Viechtbauer [9] and report the empty confidence set. As for the confidence intervals obtained using generalised Cochran heterogeneity statistics described above, providing the interval [0,0] increases the coverage probability by *α*/2 when *τ*^2^=0 but this avoids the difficulty in interpreting empty confidence sets.

If instead $Q(0)\geq \chi_{\left(n-p,\alpha /2\right)}^{2}$ then there are no further difficulties. Furthermore if $Q(0)\leq \chi_{\left(n-p,1-\alpha /2\right)}^{2}$ then $Q(\tau^{2})\leq \chi_{\left(n-p,1-\alpha /2\right)}^{2}$ for all *τ*^2^, so that the confidence interval is comprised of the *τ*^2^ that satisfy $Q(\tau^{2})\geq \chi_{\left(n-p,\alpha /2\right)}^{2}$. The lower bound of the confidence interval is then zero and the upper bound is given by the value of *τ*^2^ that satisfies $Q(\tau^{2})=\chi_{\left(n-p,\alpha /2\right)}^{2}$.

Finally, if $Q(0)>\chi_{\left(n-p,1-\alpha /2\right)}^{2}$, then the lower and upper bounds of the confidence interval are given by the values of *τ*^2^ satisfying $Q(\tau^{2})=\chi_{\left(n-p,1-\alpha /2\right)}^{2}$ and $Q(\tau^{2})=\chi_{\left(n-p,\alpha /2\right)}^{2}$ respectively. Since *Q*(*τ*^2^) is continuous and decreasing in *τ*^2^, simple numerical search methods for finding the values of *τ*^2^ that satisfy the equations that give rise to the confidence intervals will always converge to the correct answers.

#### A Newton Raphson procedure for implementing the *Q* profile method in practice

In order to implement the *Q* profile method, we need to solve equations of the form *Q*(*τ*^2^)=*c*, where *c* are critical values from $\chi_{n-p}^{2}$. Since in (13) we have the derivative of *Q*(*τ*^2^) in easily computed form we can use the Newton Raphson procedure for this purpose, where we use the iterative method

$$\tau_{k+1}^{2}=\tau_{k}^{2}+\frac{\overset{n}{\underset{i=1}{\sum }}\frac{{\left\{Y_{i}-{ŷ}_{i}\left(\hat{\beta }\left(\tau_{k}^{2}\right)\right)\right\}}^{2}}{\sigma_{i}^{2}+\tau_{k}^{2}}-c}{\overset{n}{\underset{i=1}{\sum }}\frac{{\left\{Y_{i}-{ŷ}_{i}\left(\hat{\beta }\left(\tau_{k}^{2}\right)\right)\right\}}^{2}}{{\left(\sigma_{i}^{2}+\tau_{k}^{2}\right)}^{2}}}$$

with a suitable starting value $\tau_{0}^{2}$. Once $\tau_{k+1}^{2}\approx \tau_{k}^{2}$ convergence is reached and the solution to *Q*(*τ*^2^)=*c* is obtained. This algorithm has been found to perform well provided that estimates of *τ*^2^ are used as starting values and any negative values of $\tau_{k}^{2}$ are replaced by zero when performing the iteration.

#### A corresponding point estimate of the residual between-study variance

Paule-Mandel [13,22] suggested solving $Q({\hat{\tau }}_{\text{PM}}^{2})=n-1$ in the context of meta-analysis. This idea can be generalised to the meta-regression scenario considered here [12] by solving $Q({\hat{\tau }}_{\text{PM}}^{2})=n-p$. This equation can be solved using the Newton Raphson procedure suggested above, with *c*=*n*-*p*, and if there is no solution to this equation then ${\hat{\tau }}_{\text{PM}}^{2}=0$[13,22]. The empirical Bayes estimator [23] is equivalent to this Paule-Mandel estimator [24]. By adopting the convention that empty sets are interpreted as [0,0], this estimator will always lie in the corresponding *Q* profile confidence interval. In general point estimates of *τ*^2^ need not lie inside the various confidence intervals that are available, so this is an attractive property. Hence the Paule-Mandel estimator provides a natural point estimate to accompany the confidence interval from the *Q* profile method and we recommend using them together. The Paule-Mandel estimator is also unique, as previously noted [12].

### Bayesian analyses with informative prior distributions for the residual between study variance

In addition to specifying the form of the likelihood, Bayesian analyses also require the specification of prior distributions. ‘Vague’ or ‘uninformative’ prior distributions, that are typically taken to be uniform distributions on a particular scale, are often used for this purpose. However choosing ‘vague’ prior distributions for variance components is problematic as Lambert *et al.*[25] show for the between-study variance in random effects meta-analysis with small numbers of studies. We anticipate that that the issues surrounding the use of ‘vague’ priors for variance components, as exemplified by the simulation study by Lambert *et al.*, will also apply in the meta-regression scenario.

Informative prior distributions for parameters in random effects meta-analyses are now available for some settings [10,26-28]. In practice we suggest also using prior distributions for the between-study variance that have been derived for meta-analysis as prior distributions for the residual between-study variance in meta-regressions. Deriving prior distributions for *τ*^2^ for specific meta-regression models is, at best, very difficult because there are very many combinations of study covariates that could be used in regression models. Typically meta-regression models involve only a few covariates and, although we might anticipate the residual between-study variance to be less than the between-study variance in a corresponding random effects meta-analysis (because we might expect the covariates to explain some heterogeneity), unless the covariate effects are strong we can reasonably expect the difference between these two types of between-study variance to be small. Furthermore, if the residual between-study variance is indeed the smaller of these two types of between-study variances, then using priors elicited in the context of meta-analysis for meta-regression models can be thought of as being conservative. This is because the values of *τ*^2^ supported by the prior, and hence the posterior, will be too large. This generally results in wider credible intervals and larger posterior standard deviations for the regression parameters, and favours the side of concluding that there is further unexplained variation that could be explained using additional covariates.

We follow Turner *et al.*[10] in using vague priors for the regression parameters (the entries of *β*) and informative priors for *τ*^2^. An informative prior for *τ*^2^, where available, is to be taken from the context of the meta-regression. Hence we only use out-of-sample information about the residual between-study variance, because it is this particular parameter that is hard to identify in typically small meta-analytic samples. We provide WinBUGS [29] code, for both examples, in the Additional files 1, 2 and 3 to show how informative priors for *τ*^2^ can be used in practice. The use of MCMC to fit meta-regression models means that convergence of the chains must be carefully checked in practice. Alternative (non MCMC) methods for performing Bayesian estimation of meta-regression models deserve investigation and could form the subject of further work. Meta-analysts should be aware that Bayesian analyses of this kind make more assumptions than frequentist meta-analyses because they involve priors. However, by taking the limits of the credible interval as the 2.5% and 97.5% quantiles of the posterior distribution, Bayesian interval estimation is easily performed.

## Ethical approval

This is a paper on statistical methods. All data used are for illustration purposes and come from published meta-analyses from the Cochrane Library. Hence no ethical approval for the use of these data is required.

## Results and discussion

In this section we apply our methods to two contrasting examples and we discuss the results that we obtain.

### Example one: paracetamol for pain relief after surgical removal of lower wisdom teeth

This example is taken from Analysis 1.1 of Cochrane review CD004487 [30], where paracetamol is compared to placebo and the outcome of interest is at least 50% pain relief at 4 hours. 16 studies contribute to the analysis where 10 studies treat patients with up to 1000mg of paracetamol and the remaining 6 studies treat patients with 1000mg or more. We follow the Cochrane review by using the log relative risk as the outcome measure for the analysis (the review presents the results in terms of the relative risk but the analysis is performed on the log scale), where 1/2 is added to all cells of the 2×2 tables of the two studies with zero counts. Normal approximations are used for the log relative risks.

The results of the subgroup analysis presented by the Cochrane review suggest that treatment effects may be larger in studies that treat patients with 1000mg or more. Meta-regression can be used to investigate this, by including a binary covariate that equals one if the study treats patients with 1000mg or more and equals zero otherwise. Fitting this meta-regression using the *R* package *metafor* using REML confirms that there is evidence of a covariate effect, where the estimated effect is 0.929 with a standard error of 0.302. The REML estimated residual between-study variance is (${\hat{\tau }}^{2}=0.083$), for which *metafor* reports a standard error of $\text{S.E.}({\hat{\tau }}^{2})=0.086$ and *I*^2^=40%.

Since the distribution of estimates of *τ*^2^ can be very skewed, an approximate confidence interval was calculated on the log scale using the approximation

$$\text{Var}\;\left(\text{log}\;\left({\hat{\tau }}^{2}\right)\right)\approx \frac{1}{{\hat{\tau }}^{4}}{\left(\text{S.E.}\;\left({\hat{\tau }}^{2}\right)\right)}^{2}$$

so that an approximate 95% REML confidence interval for *τ*^2^ was obtained as $\left(\exp\;\left(\log\;\left({\hat{\tau }}^{2}\right)-1.96\sqrt{\text{Var}\;(\text{log}({\hat{\tau }}^{2}))}\right),\exp\left(\log\;({\hat{\tau }}^{2})+1.96\sqrt{\text{Var}(\text{log}({\hat{\tau }}^{2}))}\right)\right)=(0.011,0.633)$. The width of this approximate confidence interval, from the asymptotic theory of maximum (REML) likelihood, gives a useful benchmark to compare the proposed methods to. We can however anticipate that the two proposed frequentist methods will result in wider confidence intervals than this interval for two main reasons. First of all, the proposed methods are not based on sufficient statistics (while likelihood based methods are). Hence we can anticipate some loss of precision when using the proposed procedures because they have not been shown to possess any optimality properties. Secondly, the asymptotic theory of maximum likelihood cannot be very accurate, because we estimate three parameters (the intercept, the covariate effect and the residual between-study variance) using just 16 observations. Since likelihood based methods take the standard errors as those implied by the Cramer-Rao bound, we can anticipate that the uncertainty in the estimated residual between-study variance will be underestimated using asymptotic likelihood based methods.

#### Confidence intervals using generalised Cochran heterogeneity statistics

Using the conventional weights $a_{i}=w_{i}=\sigma_{i}^{-2}$, the moments estimate of *τ*^2^ from (9) is 0.115 and the corresponding 95% confidence interval, obtained using Farebrother’s algorithm and numerical searches, is (0.003, 0.691). This confidence interval is in good agreement with the approximate REML interval but is slightly wider, as anticipated.

Jackson [8] suggested using the weights $a_{i}=\sigma_{i}^{-1}$ in situations where heterogeneity is anticipated but it is uncertain how much. However, this results in a notably larger ${\hat{\tau }}^{2}=0.261$, which helps to explain why the corresponding 95% confidence interval of (0.000, 1.125) is considerably wider. This choice of weights has not resulted in a shorter confidence interval for *τ*^2^ for this example.

#### Confidence intervals using the *Q* profile method

The *R* package *metafor* was used to implement the *Q* profile method. To check numerically that all the proposed methods for calculating the proposed extension of the Paule-Mandel estimator agree, this was obtained in three ways: using the *metafor* package and the empirical Bayes option, using the code provided in the Appendix of Panityakul *et al.*[12], and also using the proposed Newton Raphson procedure with *c*=14. All three methods gave ${\hat{\tau }}_{\text{PM}}^{2}=0.219$. The *Q* profile 95% confidence interval is (0.002, 1.487) which is even wider than the intervals obtained using other methods and provided above.

To summarise the results using the frequentist methods, wide confidence intervals for *τ*^2^ are obtained using all methods. This means that it is not possible to make strong statements about whether the true residual between-study variance is large or small without making use of out-of-sample information, such as expert opinion or the informative prior distributions that follow.

#### Bayesian analyses using informative prior distributions

Uninformative uniform priors (from -10 to 10) were used for the regression parameters (the entries of *β*) and an informative *τ*^2^∼logN(-1.83,1.52^2^) prior was used, as suggested by Turner *et al.*[27], where ‘logN’ denotes a log-normal distribution. Three chains were run using starting values equal to the REML estimate of *τ*^2^ and the lower and upper bounds of the corresponding approximate 95% confidence interval. A burn-in of 10,000 iterations was used and another 200,000 iterations were used to make inference, so that with three chains 600,000 simulated realisations from the posterior were used to calculate estimates and credible intervals. The WinBUGS implementation of the Gelman-Rubin statistic, as modified by Brooks and Gelman [31], was stable for each parameter and trace plots after the burn-in showed no apparent trend. These findings indicate that convergence was achieved for all three chains.

Similar results for the covariate effect parameter were obtained from this Bayesian analysis: the posterior mean was 0.917 with a posterior standard deviation of 0.330, again suggesting that treatment effects may be larger in studies that treat patients with 1000mg of paracetamol or more. The posterior distribution of *τ*^2^ was positively skewed, with a posterior mean and median of 0.135 and 0.095 respectively. The bounds of the 95% credible interval for *τ*^2^, obtained as the 2.5% and 97.5% quantiles of the posterior density, were 0.011 and 0.495. The posterior median of *τ*^2^ is quite close to the REML estimate 0.083 but the upper bound of this credible interval is appreciably less than all the upper bounds of the 95% confidence intervals. The use of this informative prior distribution has substantively reduced the range of plausible values of *τ*^2^, but this more precise inference comes at the price of specifying priors and hence making more assumptions.

A summary of the main results for this example is shown in Table 1. In order to assess the sensitivity of the estimates and credible intervals for *τ*^2^ to its prior distribution, three further priors were considered: (1) an informative *τ*^2^∼logN(-2.56,1.74^2^) for a more general research setting [10], (2) a vague uniform prior for *τ* and (3) a vague half normal prior for *τ*. See the Additional files 1, 2 and 3 for the WinBUGS code that provides full details of these alternative prior distributions. The posterior means of *τ*^2^ were 0.102, 0.188, and 0.184, and the 95% credible intervals were (0.004, 0.409), (0.002, 0.796), and (0.002, 0.779), using these three priors, respectively. We conclude that there is some sensitivity to the prior distribution, in particular the results using informative priors differ quite considerably to the results using vague priors.

**Table 1:** Summary of results for example one: paracetamol for pain relief

**Method**	**Estimate**	**Interval**
REML	0.083	(0.011, 0.633)
Gen Q ($a_{i}=\sigma_{i}^{-2}$)	0.115	(0.003, 0.691)
Gen Q ($a_{i}=\sigma_{i}^{-1}$)	0.261	(0.000, 1.125)
Paule-Mandel/Q profile	0.219	(0.002, 1.487)
Bayesian	0.135	(0.011, 0.495)

The REML confidence interval for *τ*^2^ is approximate and the Bayesian estimate is the posterior mean.

### Example two: interventions for promoting physical activity

This example is taken from Analysis 1.1 of Cochrane review CD003180 [32] and was also used by Baker and Jackson [33] who propose a novel model and estimation methods for meta-analysis datasets with time trends. Here we use a conventional meta-regression to investigate the possibility of time trends in the 19 studies that contribute to this analysis, where we use the publishing date as a continuous covariate in a meta-regression. The treatment is an intervention for promoting physical activity and the outcome data are standardised mean differences, where a positive mean difference indicates that the intervention has a positive effect on promoting physical activity.

Again fitting the meta-regression using the *metafor* package using REML provides evidence of a covariate effect and hence the type of time trend observed by Baker and Jackson [33]: the estimated effect of time is -0.022 with a standard error of 0.011. The REML estimated residual between-study variance is ${\hat{\tau }}^{2}=0.042$, for which *metafor* reports a standard error of $\text{S.E.}({\hat{\tau }}^{2})=0.021$ and *I*^2^=79%. An approximate 95% REML confidence interval for *τ*^2^, calculated on the log scale as for the previous example, is (0.016, 0.111).

#### Confidence intervals using generalised Cochran heterogeneity statistics

Using the conventional weights $a_{i}=w_{i}=\sigma_{i}^{-2}$, the moments estimate of *τ*^2^ from (9) is 0.043 to three decimal places and the corresponding 95% confidence interval is (0.017, 0.139). Once again, this confidence interval is in good agreement with the approximate REML interval but is slightly wider, as anticipated.

Using the weights $a_{i}=\sigma_{i}^{-1}$ results in similar results for this example: ${\hat{\tau }}^{2}=0.048$, and the corresponding 95% confidence interval is (0.018, 0.138).

#### Confidence intervals using the *Q* profile method

The proposed extension of the Paule-Mandel estimator is ${\hat{\tau }}_{\text{PM}}^{2}=0.049$. The *Q* profile 95% confidence interval is (0.018 0.156) which is slightly wider than the intervals obtained using other methods and provided above.

To summarise the results using the frequentist methods, wide confidence intervals for *τ*^2^ are again obtained using all methods. However, the intervals do not include zero, which reinforces the notion that residual between-study heterogeneity is present, as suggested by the large *I*^2^=79*%* statistic. Nonetheless, the true residual between-study variance could be considerably smaller or larger than the point estimate. It is not possible to make very precise statements about the magnitude of this parameter without using out-of-sample information.

#### Bayesian analyses using informative prior distributions

An informative *τ*^2^∼lt_5_(-3.02,2.27^2^) prior was used, as suggested by Rhodes *et al.*[28], where ‘ lt_5_’ denotes a log-t distribution with 5 degrees of freedom. The same uniform priors for the regression parameters, and the same numbers of chains and iterations and methods for checking convergence, were used as in the previous example. However we (approximately) standardised the time covariate in the meta-regression, by centering this at 1998 and dividing by 6, as often suggested in order to aid mixing in MCMC algorithms. Inferences in terms of the non-standardised time covariate were then obtained from the MCMC output by dividing results for the standardised covariate effect parameter by 6. This resulted in an estimated (posterior mean) effect of time of -0.022 with a posterior standard deviation of 0.011, in agreement with the REML analysis to three decimal places. The posterior distribution of *τ*^2^ was positively skewed, with a posterior mean and median of 0.050 and 0.043 respectively. The bounds of the 95% credible interval for *τ*^2^ were 0.017 and 0.119. The posterior median of *τ*^2^ is close to the REML estimate 0.042 but the upper bound of this interval is notably lower than the upper bounds of the three exact 95% confidence intervals. Interestingly, it is similar to the upper bound of the approximate REML interval, which is likely to understate the uncertainty in the residual between-study variance as explained above. However, in the case of the credible interval, it seems reasonable to conclude that the reduction of the upper bound is a result of the informative prior distribution that has, as in the first example, reduced the range of plausible values of *τ*^2^.

A summary of the main results for this example is shown in Table 2. In order to assess the sensitivity of the estimates and credible intervals for *τ*^2^ to its prior distribution, three further priors were considered: (1) an informative *τ*^2^∼lt_5_(-3.44,2.59^2^) for a more general research setting (2) a vague uniform prior for *τ* and (3) a vague half normal prior for *τ*. The posterior means of *τ*^2^ were 0.049, 0.069, and 0.071, and the 95% credible intervals were (0.016, 0.118), (0.024, 0.163), and (0.025, 0.167), using these three priors, respectively. As in the previous example, we conclude that there is some sensitivity to the prior distribution. Also as before, the results using informative priors differ quite considerably to the results using vague priors.

**Table 2:** Summary of results for example two: Interventions for promoting physical activity

**Method**	**Estimate**	**Interval**
REML	0.042	(0.016, 0.111)
Gen Q ($a_{i}=\sigma_{i}^{-2}$)	0.043	(0.017, 0.139)
Gen Q ($a_{i}=\sigma_{i}^{-1}$)	0.048	(0.018, 0.138)
Paule-Mandel/Q profile	0.049	(0.018, 0.156)
Bayesian	0.050	(0.017, 0.119)

The REML confidence interval for *τ*^2^ is approximate and the Bayesian estimate is the posterior mean.

## Conclusions

We have presented three methods for performing interval estimation of the residual between-study variance in random effects meta-regression models. All three methods are relatively straightforward extensions of methods that have been proposed for random effects meta-analysis, where there are no covariates. R code is provided in the Additional files 1, 2 and 3 for implementing the method based on generalised Cochran heterogeneity statistics but this methodology and code is now incorporated in the *metafor* package. R code for use with the *metafor* package is also provided Additional files 1, 2 and 3. The *Q* profile method has previously been described by others, so our contribution regarding this methodology is to provide a simple and easily computed form of the derivative (13) that facilitates a Newton-Raphson procedure for obtaining the confidence interval bounds. In our two examples, the use of informative prior distributions was useful in producing credible intervals with smaller upper bounds than the confidence intervals. This more precise estimation comes at the price of having to specify a prior distribution and so makes more assumptions based on out-of-sample information.

Network meta-analysis is becoming increasingly popular and a variety of approaches to modelling the variance components have been proposed in this setting [34-37]. When all studies are two arm trials, models for network meta-analysis can be expressed as standard univariate meta-regressions [38]. This means that the methods developed here are immediately relevant and applicable to network meta-analysis. Our work may therefore also serve to motivate the use of informative prior distributions for *τ*^2^ in network meta-analysis. See Xiong *et al.*[39] for an example of a network meta-analysis where informative priors are used in this way.

Interpreting point estimates of the residual between-study variance without any regard to its uncertainty can be misleading; for example, a large point estimate might be construed as providing strong evidence of considerable unexplained between-study heterogeneity. However, if the intervals we suggest contain small values of *τ*^2^ then such a conclusion is, at best, weak. Presenting standard errors (or posterior standard deviations) of *τ*^2^ is an alternative to presenting intervals but in our experience confidence and credible intervals for this parameter are generally very asymmetric. Hence we suggest that the methods for interval estimation that we have proposed are preferable, because an estimate and a standard error (or a posterior standard deviation) may give a poor indication of the range of values of *τ*^2^ supported by the data. In any case, accompanying a point estimate with some measure of its uncertainty, such as a standard error, is still preferable to giving no indication at all of the uncertainty in ${\hat{\tau }}^{2}$.

Approximate methods for constructing confidence intervals for the between-study variance in meta-analysis are available [9] and extending these to meta-regression may form the subject of future work. Since the proposed confidence intervals are not based on sufficient statistics, and have not been shown to possess any optimality properties, a comparison of their width with confidence intervals obtained using the asymptotic theory of maximum likelihood gives an indication of the loss of precision that may have been incurred by using these methods. We illustrate this comparison using both our example datasets.

Those who would wish to test the null hypothesis *H*_0_:*τ*^2^=0 versus *H*_1_:*τ*^2^>0 can obtain the results from this test by determining if the proposed confidence intervals contain zero or not. However they would report a significance level of *α*/2 when inverting these *two-sided confidence intervals* because inverting a two-sided confidence interval with coverage probability (1-*α*) gives the results of a *one-tailed test* with significance level *α*/2. *I*^2^ statistics are also very popular and can be obtained for a meta-regression by defining the “typical” within-study variance as $\sigma_{t}^{2}=(n-p)/\text{tr}(B)$, where the standard weights *A*=*W* are used. Confidence intervals for *I*^2^ can then be obtained by interpreting *I*^2^ as the monotonic function of *τ*^2^, $I^{2}=\tau^{2}/(\sigma_{t}^{2}+\tau^{2})$.

To summarise, our methods for performing interval estimation of the residual between-study variance in meta-regression models are almost as easily performed as the corresponding methods for random effects meta-analysis. We propose that confidence and credible intervals, such as those that we describe, should be routinely used when reporting the results from both meta-analyses and meta-regressions.

## Appendix 1

From the text in the Methods section we have

$$Q_{a}=Y^{t}BY=\text{tr}(Y^{t}BY)$$

Interchanging the expectation and trace operators, and using the property that tr (***A*** ***B***)=tr (***B*** ***A***), we have that

$$E(Q_{a})=\text{tr}(BE(YY^{t}))=\text{tr}(B(\Delta +\tau^{2}I+X\beta {\left(X\beta \right)}^{t}))$$

It is straightforward to show that all entries of ***B*** ***X***, and hence ***B*** ***X*** ***β*** (***X*** ***β***)^*t*^, are zero. Hence

$$E(Q_{a})=\text{tr}(B\Delta )+\text{tr}(B)\tau^{2}$$

as stated.

## Appendix 2

Let ${\hat{\beta }}_{i}(\tau^{2})$ denote the *i* entry of $\hat{\beta }(\tau^{2})$. Then we can write

$$\;Q\;(\tau^{2})=\overset{n}{\underset{i=1}{\sum }}\frac{{\left\{Y_{i}-{ŷ}_{i}\left({\hat{\beta }}_{1}\left(\tau^{2}\right),{\hat{\beta }}_{2}\left(\tau^{2}\right),\cdots \;,{\hat{\beta }}_{p}\left(\tau^{2}\right)\right)\right\}}^{2}}{\sigma_{i}^{2}+\tau^{2}}$$

Differentiating *Q*(*τ*^2^) with respect to *τ*^2^ using the product rule, and using the chain rule for multivariate calculus to evaluate the second term involving a double summation, gives

(14) $$\begin{array}{c} \frac{\text{dQ}\;(\tau^{2})}{d\tau^{2}}=-\overset{n}{\underset{i=1}{\sum }}\frac{{\left\{Y_{i}-{ŷ}_{i}\left(\hat{\beta }\left(\tau^{2}\right)\right)\right\}}^{2}}{{\left(\sigma_{i}^{2}+\tau^{2}\right)}^{2}}\\ \;-2\overset{p}{\underset{j=1}{\sum }}\frac{d{\hat{\beta }}_{j}}{d\tau^{2}}\overset{n}{\underset{i=1}{\sum }}\frac{\left\{Y_{i}-{ŷ}_{i}\left(\hat{\beta }\left(\tau^{2}\right)\right)\right\}}{\sigma_{i}^{2}+\tau^{2}}\frac{\partial {ŷ}_{i}}{\partial {\hat{\beta }}_{j}} \end{array}$$

Now consider the procedure for obtaining the estimated regression parameters for a fixed value of *τ*^2^, $\hat{\beta }(\tau^{2})$, using either, and equivalently, maximum likelihood or weighted least squares. We therefore optimise

(15) $$\ell =\overset{n}{\underset{i=1}{\sum }}\frac{{\left\{Y_{i}-{ŷ}_{i}(\beta_{1},\beta_{2},\cdots \;,\beta_{p})\right\}}^{2}}{\sigma_{i}^{2}+\tau^{2}}$$

with respect to the regression parameters. Differentiating (15) with respect to *β*_*j*_ we obtain

$$\frac{\partial \ell }{\partial \beta_{j}}=-2\overset{n}{\underset{i=1}{\sum }}\frac{\left\{Y_{i}-{ŷ}_{i}(\beta_{1},\beta_{2},\cdots \;,\beta_{p})\right\}}{\sigma_{i}^{2}+\tau^{2}}\frac{\partial {ŷ}_{i}}{\partial \beta_{j}}$$

Setting this derivative to zero implies that the second term in (14) involving a double summation is zero. This immediately results in (13).

## Competing interests

The authors declare that they have no competing interests.

## Authors’ contributions

DJ performed the statistical analyses and developed the theory for the generalised *Q* statistics and the Newton Raphson procedure for implementing the *Q* profile method. WV originally proposed the extension of the generalised *Q* statistic for calculating confidence intervals for meta-regression, developed the theory for the *Q* profile method and is the author of the *metafor* R package. VW prepared the Additional files 1, 2 and 3 document that provides code that can be used with the *metafor* package. KR and RT provided WinBUGS code that DJ adapted and provided the priors for the Bayesian analyses. KR double checked the results from the Bayesian computation. All authors contributed to the writing of the paper. All authors read and approved the final manuscript.

## Pre-publication history

The pre-publication history for this paper can be accessed here:

http://www.biomedcentral.com/1471-2288/14/103/prepub

## Supplementary Material

Additional file 1 R Code for Generalised Q Statistics (now incorporated into the metafor package). Click here for file

Additional file 2 WinBUGS code. Click here for file

Additional file 3 R Code for use with the metafor package. Click here for file
