# Supplementary material for: Methods for calculating confidence and credible intervals for the residual between-study variance in random effects meta-regression models
Source: BMC Med Res Methodol. 2014 Sep 6;14:103. doi: 10.1186/1471-2288-14-103 (PMC4160560; doi:10.1186/1471-2288-14-103)
Supplement: Additional file 1 — R Code for Generalised Q Statistics (now incorporated into the metafor package). [file 1471-2288-14-103-S1.docx]

**R Code for Generalised Q Statistics (now incorporated into the metafor package)**

This R code produces the results, using the generalised Cochran heterogeneity statistics, for the two examples in the paper; the datasets are also provided below. **The CompQuadForm and psych packages must be installed for this code to work!**

The function “inference” is called to produce the results, where inference is a function of six arguments. These five arguments are:

**y** , **v** and **Xmat**– the data: the outcomes, within-study variances, and design matrix.

**power** and **con** – The program allows use of weights of the form a_i=(1/(v_i+con))^power. power=1 and con=0 provides the conventional weights and power=0.5 and con=0 provides the other set of weights used in the paper. See Jackson [ref 8 of the paper] for more details.

**coverage** is the coverage probability of the interval, so coverage=0.95 is conventional.

The function returns three objects: **tau2** (the estimate of the between study-variance, **CI** (the confidence interval) and **width** (which is just the upper bound of the confidence interval minus the lower bound).

Copy and paste the code below into R to produce the results in table 2 using the proposed methodology.

Y1=c(1.25276296849537, 0.308701439675856, 0.182321556793955, 2.14006616349627, 1.42461322542203, 2.484906649788, 0.824175442966349, 0.0266682470821613, 2.44045488721717, 0.393042588109607, 1.23214368129263, 4.02372699500815, 1.60213860995249, 1.43828965623827, 1.13783300182139, 2.06949121082667)

Var1=c(0.16023166023166, 0.0217864923747277, 0.0555555555555556, 1.05392156862745, 0.527725563909774, 1.02350427350427, 0.0314717348927875, 0.128475365317471, 2.09667325428195, 0.0930555555555556, 0.172089947089947, 2.01996909225291, 0.183016983016983, 0.339875156054931, 0.33042735042735, 0.49009900990099)

X1=c(0, 0, 0, 0, 0, 0, 0, 0, 0, 0, 1, 1, 1, 1, 1, 1)

X1mat=matrix(1, nrow=16, ncol=2); X1mat[,2]=X1

Y2=c(0.54, 0.4, 0.64, 0.365, 0.835, 0.02, 0.12, 0.085, 1.18, 0.08,

0.18, 0.325, 0.06, 0.715, 0.065, 0.245, 0.24, 0.06, 0.19)

Var2=c(0.0176, 0.019, 0.0906, 0.0861, 0.0063, 0.0126, 0.0126, 0.0041, 0.0759, 0.0126, 0.0104, 0.0242, 0.0026, 0.2629, 0.0169, 0.0156, 0.0481, 0.0084, 0.0044)

X2=c(1986, 1987, 1988, 1988, 1998, 1999, 2000, 2000, 2000, 2001, 2001, 2001, 2002, 2002, 2002, 2002, 2003, 2003, 2003)

X2mat= matrix(1, nrow=19, ncol=2); X2mat[,2]=X2

library(CompQuadForm)

library(psych)

inference=function (y, v, Xmat, power, con, coverage)

{

alpha=(1-coverage)/2; n =length(y)

a = (1/(v+con))^power

A = diag(a)

B = A - A%*%Xmat%*%(solve(t(Xmat)%*%A%*%Xmat))%*%t(Xmat)%*%A

Y = matrix(y, ncol = 1)

Q = t(Y) %*% B %*% Y

Sigma = diag(v)

est=max(0, (Q-tr(B%*%Sigma))/tr(B))

S = (Sigma^0.5) %*% B %*% Sigma^0.5

lambda = Re(eigen(S)$values)

p=ncol(Xmat); remove=(n-p+1):n

testU = 1-farebrother(Q, lambda[-remove])$res

if (testU <= alpha) {lower=0; upper = 0}

else {upper=get_upper(y, v, Xmat, power, con, alpha)

testL = farebrother(Q, lambda[-remove])$res

if (testL >= alpha) lower = 0 else lower= get_lower(y, v, Xmat, power, con, alpha)}

return(list(estimate=est, CI=c(lower, upper), width=upper-lower))

}

get_lower=function (y, v, Xmat, power, con, alpha)

{

uniroot(get_lower1, c(0, 20), y = y, v = v, Xmat, power = power, con=con,

alpha = alpha)$root

}

get_lower1=function (x, y, Xmat, v, power, con, alpha)

{

a = (1/(v+con))^power

n = length(v)

A = diag(a)

B= A - A%*%Xmat%*%(solve(t(Xmat)%*%A%*%Xmat))%*%t(Xmat)%*%A

Y = matrix(y, ncol = 1)

Q = t(Y) %*% B %*% Y

Sigma = diag(v + x)

S = (Sigma^0.5) %*% B %*% Sigma^0.5; p=ncol(Xmat); remove=(n-p+1):n

lambda = Re(eigen(S)$values)

farebrother(Q, lambda[-remove])$res - alpha

}

get_upper=function (y, v, Xmat, power, con, alpha)

{

uniroot(get_upper1, c(0, 1000), y = y, v = v, Xmat=Xmat, power = power, con=con,

alpha = alpha)$root

}

get_upper1=function (x, y, v, Xmat, power, con, alpha)

{

a = (1/(v+con))^power

n = length(v)

A = diag(a)

B = A - A%*%Xmat%*%(solve(t(Xmat)%*%A%*%Xmat))%*%t(Xmat)%*%A

Y = matrix(y, ncol = 1)

Q = t(Y) %*% B %*% Y

Sigma = diag(v + x)

S = (Sigma^0.5) %*% B %*% Sigma^0.5; p=ncol(Xmat); remove=(n-p+1):n

lambda =Re(eigen(S)$values)

(1 - farebrother(Q, lambda[-remove])$res) - alpha

}

#Results for example 1:

inference(Y1, Var1, X1mat, 1,0,0.95)

inference(Y1, Var1, X1mat, 0.5,0,0.95)

#Results for example 2:

inference(Y2, Var2, X2mat, 1,0,0.95)

inference(Y2, Var2, X2mat, 0.5,0,0.95)
