# Supplementary material for: Methods for calculating confidence and credible intervals for the residual between-study variance in random effects meta-regression models
Source: BMC Med Res Methodol. 2014 Sep 6;14:103. doi: 10.1186/1471-2288-14-103 (PMC4160560; doi:10.1186/1471-2288-14-103)
Supplement: Additional file 2 — WinBUGS code. [file 1471-2288-14-103-S2.docx]

**WinBUGS code for example 1:**

Use the data and initial values provided (3 chains) for all models, with a burn in of 10,000 and a further 200,000 iterations to make inferences, to obtain the results in the paper.

#Data

list(y=c(1.25276296849537, 0.308701439675856, 0.182321556793955, 2.14006616349627, 1.42461322542203, 2.484906649788, 0.824175442966349, 0.0266682470821613, 2.44045488721717, 0.393042588109607, 1.23214368129263, 4.02372699500815, 1.60213860995249, 1.43828965623827, 1.13783300182139, 2.06949121082667),

var=c(0.16023166023166, 0.0217864923747277, 0.0555555555555556, 1.05392156862745, 0.527725563909774, 1.02350427350427, 0.0314717348927875, 0.128475365317471, 2.09667325428195, 0.0930555555555556, 0.172089947089947, 2.01996909225291, 0.183016983016983, 0.339875156054931, 0.33042735042735, 0.49009900990099),x=c(0, 0, 0, 0, 0, 0, 0, 0, 0, 0, 1, 1, 1, 1, 1, 1))

#Initial values

list(tau2=0.011, alpha=0.62, beta=0.92)

list(tau2=0.083, alpha=0.62, beta=0.92)

list(tau2=0.633, alpha=0.62, beta=0.92)

**# Bayesian results in Table 1**

model

{

alpha~dunif(-10,10)

beta~dunif(-10,10)

tau2~dlnorm(-1.83, prior.prec)

prior.prec<-1/(1.52*1.52)

between.prec<-1/tau2

for(i in 1:16)

{

mean[i]<-alpha+beta*x[i]

theta[i]~dnorm(mean[i], between.prec)

y[i]~dnorm(theta[i], within.prec[i])

within.prec[i]<-1/var[i]

}

}

**# Informative log-normal prior for a general research setting**

model

{

alpha~dunif(-10,10)

beta~dunif(-10,10)

tau2~dlnorm(-2.56, prior.prec)

prior.prec<-1/(1.74*1.74)

between.prec<-1/tau2

for(i in 1:16)

{

mean[i]<-alpha+beta*x[i]

theta[i]~dnorm(mean[i], between.prec)

y[i]~dnorm(theta[i], within.prec[i])

within.prec[i]<-1/var[i]

}

}

**# Non-informative uniform prior for between-study std dev**

#Initial values

list(tau=0.105, alpha=0.62, beta=0.92)

list(tau=0.288, alpha=0.62, beta=0.92)

list(tau=0.796, alpha=0.62, beta=0.92)

model

{

alpha~dunif(-10,10)

beta~dunif(-10,10)

tau~dunif(0,5)

tau2<-tau*tau

between.prec<-1/(tau*tau)

for(i in 1:16)

{

mean[i]<-alpha+beta*x[i]

theta[i]~dnorm(mean[i], between.prec)

y[i]~dnorm(theta[i], within.prec[i])

within.prec[i]<-1/var[i]

}

}

**# Non-informative half-normal prior for between-study std dev**

model

{

alpha~dunif(-10,10)

beta~dunif(-10,10)

tau~dnorm(0,0.1)I(0,)

tau2<-tau*tau

between.prec<-1/(tau*tau)

for(i in 1:16)

{

mean[i]<-alpha+beta*x[i]

theta[i]~dnorm(mean[i], between.prec)

y[i]~dnorm(theta[i], within.prec[i])

within.prec[i]<-1/var[i]

}

}

**WinBUGS code for example 2:**

Use the data and initial values provided (3 chains) for all models, with a burn in of 10,000 and a further 200,000 iterations to make inferences, to obtain the results in the paper.

#Data

list(y=c(0.54, 0.4, 0.64, 0.365, 0.835, 0.02, 0.12, 0.085, 1.18, 0.08, 0.18, 0.325, 0.06, 0.715, 0.065, 0.245, 0.24, 0.06, 0.19), var=c(0.0176, 0.019, 0.0906, 0.0861, 0.0063, 0.0126, 0.0126, 0.0041, 0.0759, 0.0126, 0.0104, 0.0242, 0.0026, 0.2629, 0.0169, 0.0156, 0.0481, 0.0084, 0.0044), x=c(1986, 1987, 1988, 1988, 1998, 1999, 2000, 2000, 2000, 2001, 2001, 2001, 2002, 2002, 2002, 2002, 2003, 2003, 2003))

#Initial values

list(logtau2=-4.13)

list(logtau2=-3.15)

list(logtau2=-2.20)

**# Bayesian results in Table 2**

model

{

alpha~dunif(-10,10)

beta~dunif(-10,10)

tau2<-exp(logtau2)

prior.prec<-1/(2.27*2.27)

logtau2~dt(-3.02, prior.prec,5)

between.prec<-1/tau2

for(i in 1:19)

{

mean[i]<-alpha+beta*(x[i]-1998)/6

theta[i]~dnorm(mean[i], between.prec)

y[i]~dnorm(theta[i], within.prec[i])

within.prec[i]<-1/var[i]

}

}

**# Informative log-normal prior for a general research setting**

model

{

alpha~dunif(-10,10)

beta~dunif(-10,10)

tau2<-exp(logtau2)

prior.prec<-1/(2.59*2.59)

logtau2~dt(-3.44, prior.prec,5)

between.prec<-1/tau2

for(i in 1:19)

{

mean[i]<-alpha+beta*(x[i]-1998)/6

theta[i]~dnorm(mean[i], between.prec)

y[i]~dnorm(theta[i], within.prec[i])

within.prec[i]<-1/var[i]

}

}

**# Non-informative uniform prior for between-study std dev**

#Initial values

list(tau=0.127, alpha=0.3, beta=-0.1)

list(tau=0.207, alpha=0.3, beta=-0.1)

list(tau=0.333, alpha=0.3, beta=-0.1)

model

{

alpha~dunif(-10,10)

beta~dunif(-10,10)

tau~dunif(0,5)

tau2<-tau*tau

between.prec<-1/(tau*tau)

for(i in 1:19)

{

mean[i]<-alpha+beta*x[i]

theta[i]~dnorm(mean[i], between.prec)

y[i]~dnorm(theta[i], within.prec[i])

within.prec[i]<-1/var[i]

}

}

**# Non-informative half-normal prior for between-study std dev**

model

{

alpha~dunif(-10,10)

beta~dunif(-10,10)

tau~dnorm(0,0.1)I(0,)

tau2<-tau*tau

between.prec<-1/(tau*tau)

for(i in 1:19)

{

mean[i]<-alpha+beta*x[i]

theta[i]~dnorm(mean[i], between.prec)

y[i]~dnorm(theta[i], within.prec[i])

within.prec[i]<-1/var[i]

}

}
