# Supplementary material for: Methods for calculating confidence and credible intervals for the residual between-study variance in random effects meta-regression models
Source: BMC Med Res Methodol. 2014 Sep 6;14:103. doi: 10.1186/1471-2288-14-103 (PMC4160560; doi:10.1186/1471-2288-14-103)
Supplement: Additional file 3 — R Code for use with the metafor package. [file 1471-2288-14-103-S3.docx]

**R Code for use with the metafor package**

The R code below reproduces the results for examples one and two in the paper. In both cases, yi is a vector with the effect size estimates (log relative risks in the first example, standardized mean differences in the second example), vi is a vector with the corresponding sampling variances, and xi is a vector with the covariate/moderator values (a dummy variable to indicate the subgroup in the first example, publication year in the second example).

### need to install the 'metafor' and 'CompQuadForm' packages

### run this once in case these packages are not yet installed:

### install.packages(c("metafor", "CompQuadForm"))

library(metafor)

############################################################################

### example 1 ###

yi <- c(0.0267, 0.8242, 0.3930, 2.4405, 2.1401, 1.2528, 2.4849, 0.3087,

1.4246, 0.1823, 1.1378, 1.2321, 2.0695, 4.0237, 1.4383, 1.6021)

vi <- c(0.1285, 0.0315, 0.0931, 2.0967, 1.0539, 0.1602, 1.0235, 0.0218,

0.5277, 0.0556, 0.3304, 0.1721, 0.4901, 2.0200, 0.3399, 0.1830)

xi <- c(0, 0, 0, 0, 0, 0, 0, 0, 0, 0, 1, 1, 1, 1, 1, 1)

### random/mixed-effects meta-regression model (REML estimation by default)

res <- rma(yi, vi, mods = ~ xi, digits=3)

res

### approximate 95% CI for tau^2 based on REML estimate and its SE

round(exp(log(res$tau2) + c(-1.96,1.96)*(1/res$tau2 * res$se.tau2)),3)

### generalised Cochran heterogeneity estimate and CI (inverse variance weights)

res <- rma(yi, vi, mods = ~ xi, method="GENQ", weights=1/vi, digits=3)

confint(res)

### generalised Cochran heterogeneity estimate and CI (inverse SE weights)

res <- rma(yi, vi, mods = ~ xi, method="GENQ", weights=1/sqrt(vi), digits=3)

confint(res)

### Paule-Mandel estimate and CI

res <- rma(yi, vi, mods = ~ xi, method="PM", digits=3)

confint(res)

############################################################################

### example 2 ###

yi <- c(0.54, 0.4, 0.64, 0.365, 0.835, 0.02, 0.12, 0.085, 1.18, 0.08, 0.18,

0.325, 0.06, 0.715, 0.065, 0.245, 0.24, 0.06, 0.19)

vi <- c(0.0176, 0.019, 0.0906, 0.0861, 0.0063, 0.0126, 0.0126, 0.0041, 0.0759,

0.0126, 0.0104, 0.0242, 0.0026, 0.2629, 0.0169, 0.0156, 0.0481, 0.0084,

0.0044)

xi <- c(1986, 1987, 1988, 1988, 1998, 1999, 2000, 2000, 2000, 2001, 2001, 2001,

2002, 2002, 2002, 2002, 2003, 2003, 2003)

### random/mixed-effects meta-regression model (REML estimation by default)

res <- rma(yi, vi, mods = ~ xi, digits=3)

res

### approximate 95% CI for tau^2 based on REML estimate and its SE

round(exp(log(res$tau2) + c(-1.96,1.96)*(1/res$tau2 * res$se.tau2)),3)

### generalised Cochran heterogeneity estimate and CI (inverse variance weights)

res <- rma(yi, vi, mods = ~ xi, method="GENQ", weights=1/vi, digits=3)

confint(res)

### generalised Cochran heterogeneity estimate and CI (inverse SE weights)

res <- rma(yi, vi, mods = ~ xi, method="GENQ", weights=1/sqrt(vi), digits=3)

confint(res)

### Paule-Mandel estimate and CI

res <- rma(yi, vi, mods = ~ xi, method="PM", digits=3)

confint(res)

############################################################################
